# Durability of bioprosthetic aortic valves in patients under the age of 60 years – rationale and design of the international INDURE registry

**DOI:** 10.1186/s13019-020-01155-6

**Published:** 2020-05-27

**Authors:** Bart Meuris, Michael A. Borger, Thierry Bourguignon, Matthias Siepe, Martin Grabenwöger, Günther Laufer, Konrad Binder, Gianluca Polvani, Pierluigi Stefano, Enrico Coscioni, Wouter van Leeuwen, Philippe Demers, Francois Dagenais, Sergio Canovas, Alexis Theron, Thierry Langanay, Jean-Christian Roussel, Olaf Wendler, Giovanni Mariscalco, Renzo Pessotto, Beate Botta, Peter Bramlage, Ruggero de Paulis

**Affiliations:** 1 Cardiac Surgery, University Hospitals Leuven, Herestraat 49, 3000 Leuven, Belgium; 2 Leipzig Heart Center, Leipzig, Germany; 3 Tours University Hospital, Tours, France; 4 Heart Center University of Freiburg, Freiburg and Bad Krozingen, Germany; 5 HZH Heart Center Hietzing, Wien, Austria; 6 Medicine University Wien, Heart Center, Wien, Austria; 7 Heart Center University St. Pölten, St. Pölten, Austria; 8 Cardiology Center Monzino, Milan, MI Italy; 9 Careggi University Hospital, Florence, Italy; 10 University Hospital “San Giovanni di Dio e Ruggi d’Aragona”, Salerno, Italy; 11 Erasmus University Medical Center, Rotterdam, Netherlands; 12 Montreal Heart Institute, Montréal, Canada; 13 Institute University, Cardiology Center, Québec, Canada; 14 Hospital University Virgen de la Arrixaca, Murcia, Spain; 15 Hospital de la Timone, Marseille, France; 16 Rennes University Hospital Center, Rennes, France; 17 CHU Nantes, Nantes, France; 18 King’s College Hospital NHS Foundation Trust, London, UK; 19 Glenfield Hospital, Leicester, UK; 20 Royal Infirmary of Edinburgh, Edinburgh, UK; 21 Institute for Pharmacology and Preventive Medicine, Cloppenburg, Germany; 22 Cardiac Surgery, European Hospital, Rome, Italy

**Keywords:** Aortic valve disease, Surgical aortic valve replacement, SAVR, INSPIRIS RESILIA, Structural valve degeneration, Valve durability

## Abstract

**Background:**

There is an ever-growing number of patients requiring aortic valve replacement (AVR). Limited data is available on the long-term outcomes and structural integrity of bioprosthetic valves in younger patients undergoing surgical AVR.

**Methods:**

The INSPIRIS RESILIA Durability Registry (INDURE) is a prospective, open-label, multicentre, international registry with a follow-up of 5 years to assess clinical outcomes of patients younger than 60 years who undergo surgical AVR using the INSPIRIS RESILIA aortic valve. INDURE will be conducted across 20–22 sites in Europe and Canada and intends to enrol minimum of 400 patients. Patients will be included if they are scheduled to undergo AVR with or without concomitant root replacement and/or coronary bypass surgery.

The primary objectives are to 1) determine VARC-2 defined time-related valve safety at one-year (depicted as freedom from events) and 2) determine freedom from stage 3 structural valve degeneration (SVD) presenting as morphological abnormalities and severe haemodynamic valve degeneration at 5 years. Secondary objectives include the assessment of the haemodynamic performance of the valve, all stages of SVD, potential valve-in-valve procedures, clinical outcomes (in terms of New York Heart Association [NYHA] function class and freedom from valve-related rehospitalisation) and change in patient quality-of-life.

**Discussion:**

INDURE is a prospective, multicentre registry in Europe and Canada, which will provide much needed data on the long-term performance of bioprosthetic valves in general and the INSPIRIS RESILIA valve in particular. The data may help to gather a deeper understanding of the longevity of bioprosthetic valves and may expand the use of bioprosthetic valves in patients under the age of 60 years.

**Trial registration:**

ClinicalTrials.gov identifier: NCT03666741 (registration received September, 12th, 2018).

## Background

There is an ever-growing number of patients requiring aortic valve replacement (AVR) [1]. The two principal reasons for AVR are aortic regurgitation (AR) and aortic stenosis (AS), the latter being the most common indication. Although the majority of patients is older [2], younger patients are of particular concern as they have a longer lifespan with their replaced valve and are dependent on properly functioning, non-deteriorated valves over this much longer term.

In general, a wide spectrum of therapies can be offered to younger patients such as sparing valve techniques and mechanical valve replacement. Homografts are possible but less popular due to inferior longevity. The Ross procedure gains in popularity in selected expert centres. Mechanical valves have been preferred over bioprosthetic valves in younger patients, but this is not equivocal. While some studies have shown a survival benefit of mechanical valves in younger patients [3–7], large retrospective observational studies [8–12] and one randomized controlled trial [13] have shown similar long-term survival in patients 50 to 69 years of age undergoing mechanical versus bioprosthetic valve replacement.

Based on these data, the 2017 American Heart Association (AHA)/American College of Cardiology (ACC) guidelines on valvular heart disease [14] recommend mechanical over bioprosthetic valves in patients below the age of 50 years (class IIa, lowered from 60 years in the 2014 version) and suggest an individualised choice (so called grey-zone) of either a mechanical or bioprosthetic valve in patients between 50 and 70 years. Conversely, the 2017 European guidelines recommend the use of mechanical valves in patients under the age of 60 years unless good quality anticoagulation is unlikely and a grey zone between 60 and 65 years [15]. Both guidelines emphasize the need to consider the desire of an informed patient when it comes to the choice of the valve treatment.

The INSPIRIS RESILIA aortic Valve™ (Edwards Lifesciences, Irvine, USA) is a stented bioprosthetic, tri-leaflet valve comprised of bovine pericardial tissue. Specific new tissue preservation techniques result in a stable capping process, which blocks residual aldehyde groups known to bind calcium, in addition to a phospholipid removal process. A final tissue glycerolisation step allows valve storage without further tissue exposure to glutaraldehyde. Finally, INSPIRIS RESILIA features an expansion feature, called VFit, intended for future potential valve in valve procedures.

RESILIA tissue demonstrated, in a large pre-clinical randomized control trial conducted in juvenile sheep in mitral position, to significantly reduce tissue calcification (− 72%) and even to improve haemodynamic performance compared with the Perimount valve [16]. The RESILIA tissue also has been studied in two clinical trials to date [17, 18]. Bartus et al. found, in a single-arm observational study of 133 patients, that the RESILIA tissue provided excellent performance and safety without structural valve deterioration (SVD) [18–20]. In the COMMENCE trial [17, 20], 679 patients underwent Carpentier-Edwards PERIMOUNT Magna Ease™ aortic valve replacement with RESILIA™ tissue (Model 11000A) and similar excellent safety and effectiveness were demonstrated for up to 4 years without SVD. Both of these trials have included relatively young patients, with a mean age between 65 and 67 years and up to 26% of patients aged less than 60 years.

These trials generated useful insights on the safety and effectiveness of the RESILIA tissue, but they were not specifically designed to assess durability in younger patients which received the INSPIRIS RESILIA aortic valve and data on this topic in general is scarce [21, 22].

It is for this reason that we designed a prospective long-term registry around the INSPIRIS RESILIA aortic Valve™. With 400 patients under the age of 60 years included and a follow-up of 5 years, we will collect data on the short-term clinical effectiveness of the valve’s implantation, as well as pivotal data on the long-term haemodynamic and structural performance of the valve.

## Methods/design

The INDURE registry is a prospective, open-label, multicentre, international registry with a follow-up of 5 years to assess the clinical outcomes of patients younger than 60 years of age who undergo surgical AVR with the INSPIRIS RESILIA aortic valve (Edwards Lifesciences). The registry is conducted according to ISO 14155:2011. Approximately 400 patients will be enrolled across 20–22 European sites (including Austria, Belgium, France, Germany, Italy, the Netherlands, Spain, and UK) and Canada, resulting in about 20 patients per site. It was estimated, from the COMMENCE Trial dataset [17, 20], that freedom from time-related valve safety events at 1-year (composite endpoint according to VARC-2 criteria) is around 91.5%, suggesting that 400 patients will arrive at a 95% confidence interval (CI) of ±2.14%. Lower rates (80%) will broaden the 95% CI to ±3.92% and higher rates (99%) narrow it down to ±1%.

### Patients

Patients under the age of 60 years undergoing SAVR and receiving the INSPIRIS RESILIA aortic valve prosthesis will be enrolled on a consecutive basis. In addition to the applicable criteria of the device Instructions for Use (IFU), the registry inclusion criteria stipulate that patients require a planned replacement of their native valve as indicated in a preoperative evaluation, are scheduled to undergo planned AVR with or without concomitant root replacement and/or coronary bypass surgery. The latter is understood as isolated AVR with or without CABG and ascending aortic replacement. Also allowed is pulmonary vein isolation if it is not a full cox-maze procedure. Patients with a Bentall procedure or any surgery on other valves are not allowed in this registry. Patients need to be available to attend yearly follow-up visits at the registry centre for up to 5 years and all patients are required to provide written informed consent.

Patients will be excluded from the study if 1) they have active endocarditis/myocarditis at the time of surgery or have had it within the last 3 months of the scheduled SAVR, 2) have had previous AVR, 3) valve implantation is not possible in accordance with the device IFU, or 4) they have an estimated life expectancy of less than 12 months for any reason. The intraoperative exclusion criterion is that valve implantation is not possible in accordance with the device IFU.

### Objectives

The primary objectives (Table 1) are to 1) determine the time-related valve safety at 1-year (composite endpoint according to the VARC-2 criteria) depicted as freedom from events [23] and 2) determine freedom from stage 3 SVD following the Salaun definition at 5 years [23, 24]. Events include SVD (either valve-related dysfunction, defined by haemodynamic parameters or the need for repeat procedure), prosthetic valve endocarditis, prosthetic valve thrombosis, thromboembolic events (e.g., stroke) and valve-related bleeding.

**Table 1:** Registry objectives

**Primary objectives**	Time-related valve safety at 1 year (composite endpoint according to VARC-2 [23] depicted as freedom from the following events: • SVD (valve-related dysfunction [MPG ≥20 mmHg, EOA ≤0.9–1.1 cm^2^ and/or DVI < 0.35 m/s, AND/OR moderate or severe prosthetic valve regurgitation], requiring repeat procedure [TAVI or SAVR]) • Valve-related dysfunction • Requirement of repeat procedures (Re-intervention) • Prosthetic valve endocarditis • Prosthetic valve thrombosis • Thromboembolic events (e.g., stroke) • Valve-related VARC bleeding
	To determine freedom from stage 3 SVD following [24] at 5 years
**Secondary objectives**	Haemodynamics and durability: • Haemodynamic performance of the INSPIRIS RESILIA Aortic Valve™ including PPM • SVD following Salaun [24] • Description of potential ViV procedures and clinical outcome including follow-up
	Clinical outcomes: • NYHA functional class compared to baseline • Freedom from valve-related rehospitalisation
	Quality-of-life: • 3–6-month, 1-year and 3-year change from baseline in quality-of-life assessed by the KCCQ and SF-12 Health Survey
**Exploratory: Rehospitalisation and costs**	Length of hospital stay
	Length of time in intensive care unit
	Time to return to work
	Rate of valve-related rehospitalisation and associated costs (average costs per country)
	Rate of transfusion for bleeding and associated costs (average costs per country)
	Costs of a major bleeding event
	Costs of daily anticoagulation
	Rate of re-intervention for valve degeneration and associated costs (average costs per country)
**Exploratory: Safety**	SVD
	Non-SVD
	Thromboembolic events (e.g., stroke)
	Valve thrombosis
	All bleeding/haemorrhage
	Major bleeding/haemorrhage
	All paravalvular leak
	Major paravalvular leak
	Endocarditis
	All-cause mortality
	Cardiac-related mortality
	Valve-related mortality
	Valve-related re-intervention
	Conduction disturbances
	Myocardial infarction
	Deep sternal wound infection
	Acute kidney injury

*DVI* Doppler velocity index, *EOA* Effective orifice area, *KCCQ* Kansas City Cardiomyopathy Questionnaire, *MPG* Mean pressure gradient, *NYHA* New York Heart Association, *PPM* Patient prosthesis mismatch, *SAVR* Surgical aortic valve replacement, *SF-12* Short Form-12, *SVD* Structural valve degeneration, *TAVI* Transcatheter aortic valve implantation, *VARC* Valve Academic Research Consortium, *ViV* Valve-in-valve

The secondary objectives are designed to assess haemodynamic performance and further durability parameters, clinical outcomes and quality-of-life (QoL). The first group of objectives is further defined as the haemodynamic performance of the INSPIRIS RESILIA aortic valve including patient prosthesis mismatch (PPM); SVD following the Salaun definition; and the description of potential valve-in-valve procedures and clinical outcomes. Clinical outcomes of interest are NYHA functional class compared to baseline and freedom from valve-related hospitalisation. Quality-of-life will be assessed using the Kansas City Cardiomyopathy Questionnaire (KCCQ) and Short Form-12 Health Survey (SF-12) [24]. Various additional exploratory analyses regarding rehospitalisation, costs and safety will also be performed.

### Data collection

The clinical outcome data collected will be based on the site’s standard-of-care for surgical AVR. Data will be collected prospectively, according to the timetable set out in Table 2, and include medical history, physical assessments, electrocardiogram (ECG), laboratory results, computerised tomography (CT) scans (if performed as a standard-of-care), transthoracic/transoesophageal echocardiography and QoL measures. Anti-thrombotic therapy and medications are at the discretion of each investigator. Data will be captured on an electronic case report form (eCRF) by either a study nurse or physician, and data will be checked automatically for plausibility and completeness.

**Table 2:** Data collection schedule

	Baseline/screening	Surgery	Discharge	3–6 months^a^	Years 1–5
Signed informed consent	X				
Medical history^b^	X				
Physical examination^c^	X		X		X
ECG (12-lead)	X		X		X
Echocardiogram (TTE)	X		X		X
Core Lab echo					X^e^
MSCT (if available)					X^f^
NYHA class/CCS angina class	X			X	X
QoL questionnaire (SF-12 and KCCQ)	X			X	X^g^
Anti-thromboembolic therapy and medications	X		X	X	X
Procedural information		X			
Aetiology		X			
SAE reporting		X	X	X	X
Discharge data			X		
Rehospitalisation data^d^				X	X
Return to work				X	X

*CCS* Canadian Cardiovascular Society, *ECG* Electrocardiogram, *KCCQ* Kansas City Cardiomyopathy Questionnaire, *MSCT* Multi-slice computed tomography, *NYHA* New York Heart Association, *QoL* Quality of life, *SAE* Serious adverse event(s), *SF-12* Short Form-12 Health Survey, *TTE* Transthoracic echocardiogram

^a^Data captured over a telephone call

^b^Includes cardiovascular and non-cardiovascular conditions, prior cardiac interventions and surgeries

^c^Physical examination, includes height, weight and vital signs (blood pressure and heart rate)

^d^Includes re-interventions, potential valve-to-valve procedures, associated costs

^e^Solely performed at 1-year and 5-years of follow-up

^f^Solely documented at 5-years of follow-up

^g^Solely performed at baseline, 3–6 months and years 1 and 3 of follow-up

### Echocardiography core lab

Digital imaging and communication in medicine (DICOM) files of echocardiograms generated at years 1 and 5 will be collected for analysis by the Echo Core Laboratory to ensure unbiased and consistent analysis of the diagnostic data and, with the use of serial echocardiographic studies conducted on the same patient, for evaluating patient status over the course of 5 years.

### Statistical analysis

Continuous variables will be presented as mean ± standard deviation (SD) or as median with interquartile range (IQR), and categorical variables (e.g., gender) will be reported as frequencies and percentages. The Kolmogorov-Smirnov test will be used to test for normal distribution. Accordingly, the Student t-test or Mann-Whitney U test will be used to test for statistically significant differences. The Chi-Square or Fisher Exact test will be used for statistical distribution analysis of categorical variables. Kaplan-Meier analyses will be performed for survival and safety outcomes. Linearized rates and actuarial probability statistics will be used where appropriate for adverse event reporting. A *P*-value of < 0.05 will be considered statistically significant. Statistical analysis will be performed using SPSS Version 24.0 (Armonk, NY, IBM Corp.).

## Discussion

The INDURE registry has been designed to provide prospectively collected data that can be used to elucidate the benefits and risks of the surgical implantation of INSPIRIS RESILIA in patients with AVR who are younger than 60 years of age, as well as the long-term haemodynamic and structural performance of the valve in patients in this age group. Analysis of the data may also provide additional support for the earlier use (e.g., at a younger age) of bioprosthetic valves in patients undergoing AVR.

### Bioprosthetic vs. mechanical valves

Mechanical valves are generally preferred over bioprosthetic valves for younger patients undergoing AVR because of their perceived greater durability with the 15-year rates of redo-surgery being 6.9% for mechanical and 12.1% for biological heart valves [10]. It is suggested that mechanical valves will last throughout the remainder of the patient’s lifetime [25]. Mechanical valves do, however, require daily treatment with anticoagulants, which will increase the risk of bleeding. Lifelong anticoagulation can be difficult for patients with a history of bleeding issues or an increased risk of injury related to an active lifestyle. There may also be dietary restrictions, including reducing the intake of foods rich in vitamin K when taking vitamin K antagonists [26]. Newer (or non-vitamin K) oral anticoagulants (NOACs) are strictly contraindicated in patients with any mechanical prostheses [15, 27, 28]. Next to all the anticoagulation-related problems, reoperations can be needed even in mechanical valves in case of pannus overgrowth. Bioprosthetic valves do not require long-term daily anticoagulants but are at risk of SVD requiring reoperation [26]. The risk/benefit profile of mechanical versus bioprosthetic valves has led to both American and European guidelines on valvular heart disease recommending the use of mechanical valves in patients younger than 50 years [14, 15] with the European version extending this recommendation to patients up to 60 years (class IIa, level C) and the American guidelines considering both mechanical and bioprosthetic valves in patients between 50 and 70 years of age (class IIa, level B, no RCT data). Despite these recommendations, the use of bioprosthetic valves has significantly increased over the last few decades across all age groups [26]. Currently bioprosthetic valves are being developed that avoid the risk of valve required anticoagulation while reducing the reoperation rates seen with earlier generation bioprosthetic valves.

### Determinants and surrogates of valve failure

The ultimate goal when developing durable bioprosthetic valves, which are particularly required for younger patients, is to ascertain an uncompromised haemodynamic function over the very long term with no structural degeneration that would otherwise lead to a requirement for valve replacement or valve-in-valve (ViV) interventions or death [29, 30]. The data required, however, would take 10, 15 or even 20 years to be collected and assessed and, as such, shorter-term surrogates of valve degeneration have been developed which facilitate shorter valve development cycles. The criteria are plenty, but have been recently reviewed by different author groups including Capodanno et al. [31], Dvir et al. [32] and Salaun et al. [24], partly in an attempt to provide standardised definitions of SVD for bioprosthetic aortic valves. The definition of SVD by Salaun [24] (Table 3) has been adopted in the current project as it incorporates terminology proposed by both Dvir [32] and Capodanno [31] and was compatible with the definition used by Pibarot et al. [34] (see below). We will, however, capture the components of the other definitions as well aiming to explore and compare these as well.

**Table 3:** Definition of structural valve deterioration of aortic bioprostheses following Salaun [24]

	Stage 0^a^ No SVD	Stage 1^a^ SVD with no HVD ‘Morphological SVD’^b^	Stage 2^a^ SVD with moderate HVD ‘moderate hemodynamic SVD’^b^	Stage 3^a^ SVD with severe HVD ‘Severe hemodynamic SVD’^b^
Valve leaflet morphology and motion by TTE, TEE or MDCT				
Leaflet morphology				
Valve leaflet thickening: At least one leaflet with thickness ≥ 2 mm	Absent	Present	Present	Present
Valve leaflet fibrocalcific remodelling: Hyper echogenicity (TTE/TEE) or hyper density (MDCT) Detectable leaflet calcification at MDCT	Absent	Present	Present	Present
Leaflet motion				
Reduced mobility	Absent	Absent or mild	Mild to moderate	Moderate to severe
Leaflet tear/avulsion	Absent	Absent	May be present	May be present
Valve haemodynamics by TTE				
Mean transprosthetic gradient				
Absolute increase from baseline^c^	< 10 mmHg	< 10 mmHg	10–19 mmHg	≥20 mmHg
Mean gradient at post-AVR echo^d^	< 20 mmHg	< 20 mmHg	20–39 mmHg	≥40 mmHg
Valve effective orifice area				
Absolute decrease from baseline	< 0.30 cm^2^	< 0.30 cm^2^	0.30–0.59 cm^2^	≥0.60 cm^2^
Doppler velocity index				
Absolute decrease from baseline	< 0.1	< 0.1	0.1–0.2	≥0.2
Transprosthetic valve regurgitation^c^				
Worsening compared with baseline	Absent	None	≥1 Grade	≥2 Grades
Grade of regurgitation	None, trace or mild	None, trace or mild	Moderate	Severe
Clinical status		Subclinical	Often subclinical	Generally clinically expressive
New onset or worsening symptoms		Absent	Often absent	Generally present
New onset or worsening LV dilation/hypertrophy/dysfunction		Absent	Generally absent	Often present
New onset or worsening pulmonary hypertension		Absent	Generally absent	Often present

The classification and criteria presented in this table are based on recommendations and standardised definitions of medical societies or group of experts [31–33]

Stage 0 refers to a normal valve. Stage 1 consists in the presence of morphological abnormalities of valve leaflets but with no evidence of HVD. At echocardiography, the leaflets are thickened (> 2 mm), often irregular and hyperechogenic. MDCT without contrast may be used to detect and quantitate macroscopic valve leaflet calcification by the modified Agatston method or the volumetric method. Stage 2 consists in SVD with moderate HVD defined as: (1) an increase in mean transprosthetic gradient ≥10 mmHg since early post-SAVR or TAVI echocardiography with concomitant decrease in valve EOA and DVI; and/or (2) a new onset or worsening of transprosthetic regurgitation by at least one grade with a final grade of moderate. An increase in transprosthetic velocity and gradients with concomitant increase in valve EOA and DVI is actually related to an increase in flow during follow-up and should not be mistaken for an HVD. Stage 3 consists in SVD with severe HVD characterised by: (1) an increase in mean transprosthetic gradient ≥20 mmHg since SAVR or TAVI with concomitant marked decrease in valve EOA and DVI and/or (2) new onset or worsening of transprosthetic regurgitation by at least two grades with final grade of severe regurgitation

*AVR* Aortic valve replacement, *DVI* Doppler velocity index, *EOA* Effective orifice area, *HVD* Haemodynamic valve deterioration, *LV* Left ventricle, *MDCT* Multidetector CT, *SVD* Structural valve deterioration, *SAVR* Surgical aortic valve replacement, *TAVI* Transcatheter aortic valve implantation, *TEE* Transesophageal echocardiography, *TTE* Transthoracic echocardiography

Classification terminology proposed by: ^a^Dvir et al. [32] and ^b^Capodanno et al. [31]

^c^The most important criteria to define haemodynamic HVD is a significant increase in mean transprosthetic gradient with concomitant decrease in valve EOA and DVI; and/or a new onset or a worsening of transprosthetic valve regurgitation

^d^This criterium is corroborative but should not be used in isolation to define HVD

### INDURE in perspective

The RESILIA tissue has been studied in two trials to date [17, 18] with a total of 812 patients showing that its use results in excellent haemodynamic performance and safety up to 2 years. Both the RESILIENCE trial [34] and the INDURE registry were set up in order to assess the long-term performance and structural integrity of bioprosthetic valves using the RESILIA tissue in younger patients. RESILIA tissue incorporates an anti-calcification process, by permanently blocking the residual aldehyde groups that are known to bind calcium. Calcification is known to occur more commonly on bioprosthetic valves than mechanical valves [35]. It, therefore, has the potential to increase valve longevity and consequently reduce re-intervention rates.

Both INDURE and RESILIENCE (Table 4) are prospective studies, including patients with either the INSPIRIS RESILIA valve (INDURE) or any RESILIA tissue bearing valve (RESILIENCE). While INDURE will follow patients from the time of surgery for 5 years, RESILIENCE pursues retrospective inclusion of patients with the first visit being 5-years after surgical intervention and a prospective follow-up (up to year 11 after the implant). On the one hand, INDURE puts emphasis on a combination of time-related valve safety at 1-year, SVD defined according to Salaun [24] using a CoreLab and clinical outcomes, while on the other hand RESILIENCE focuses on the multi-slice computed tomography (MSCT) and echo-based (both CoreLab) prediction of re-intervention or valve-related death. Projected completion dates are 2025 (INDURE) and 2027 (RESILIENCE), respectively.

**Table 4:** INDURE vs. RESILIENCE

	INDURE (NCT03666741)	RESILIENCE (NCT03680040)
Valve used	INSPIRIS valves	RESILIA tissue valves
Design	Prospective	Retrospective inclusion, prospective follow-up
Study Start date	26 April 2019	5 November 2018
Baseline	Implantation	5 years
Follow-up	5 years – projected completion 2025	6 years (from year 5 to year 11) – projected completion 2027
Subjects/centres	400 subjects, 20–25 centres (EU and Canada) under the age of 60 years at the time of their SAVR	220 subjects, up to 15 centres (US and EU) under the age of 65 years at the time of their SAVR
Objective	Assess clinical outcomes	Time to valve failure due to valve degeneration requiring re-intervention & early potential predictors of valve durability
Primary endpoints	Time-related valve safety at 1 year (VARC-2)	Time to BVF due to SVD, defined as requiring re-intervention (redo surgery or ViV), or confirmed valve related death, according to Akin criteria [29]
	Rate of severe SVD (stage 3 following Salaun [24]) at 5 years (Echo CoreLab)	
Secondary endpoints	Haemodynamics and durability (Echo CoreLab) Clinical outcomes (NYHA and freedom from rehospitalisation) Quality-of-life (KCCQ & SF-12)	Early possible predictors of valve failure including leaflet calcification and morphological/haemodynamic valve degeneration: -Valve leaflet calcification via CoreLab evaluated MSCT (no contrast) -Haemodynamic performance (Echo CoreLab)

*BVF* Bioprosthetic valve failure, *EU* European Union, *KCCQ* Kansas City Cardiomyopathy Questionnaire, *MSCT* Multi-slice computed tomography, *NYHA* New York Heart Association, *SF-12* Short Form-12, *SVD* Structural valve degeneration, *US* United States, *VARC* Valve Academic Research Consortium, *ViV* Valve-in-valve

Up and beyond INDURE and RESILIENCE there is a third long-term data collection ongoing (IMPACT; NCT04053088) using the INSPIRIS RESILIA valve. It is being conducted in Germany, Austria, Switzerland and The Netherlands and will follow up to 500 patients for 5 years. The principal objective of IMPACT is the assessment of the impact of comorbidities such as chronic kidney disease (CKD), diabetes, hypertension, metabolic syndrome and inflammation on all-cause mortality. Among the secondary objectives there is, again, assessment of SVD, which will complement the data derived from INDURE and RESILIENCE.

### Appreciation of the study design

The INDURE registry is a prospective, open-label, multicentre, international registry. The multinational nature of this registry increases the applicability of findings to clinical practice all over Europe and Canada. However, it has no control group making a comparison of different bioprosthetic valves or valve generations impossible. Furthermore, there is no comparison of the bioprosthetic valve data with the outcomes of mechanical valve implantation, which would be desirable, but goes beyond the possibilities of such a project. Because of the multicentre design, an Echo CoreLab has been established to have a uniform assessment of SVD over the time course of the 5-year follow-up. We considered establishing an MSCT CoreLab, as has been incorporated in the RESILIENCE trial, but it would have violated the non-interventional nature of the INDURE registry as most sites reported that an MSCT is not standard-of-care at their institution, which may be considered as a limitation. Nonetheless these data can be documented in case they are available from routine practice. Finally, the same INSPIRIS RESILIA valve will be used in all patients in the INDURE registry which will abolish any bias introduced by the use of different bioprosthetic valves.

## Conclusions

INDURE is a prospective, multicentre registry in Europe and Canada that will provide much needed data on the long-term durability of bioprosthetic valves in general and the INSPIRIS RESILIA valve in particular. The data may help to gather a deeper understanding of the longevity of bioprosthetic valves and may expand the use of bioprosthetic valves in patients under the age of 50 and 60 years.

## Data Availability

Not applicable.

## Abbreviations

ACC: American College of Cardiology
AHA: American Heart Association
AS: Aortic stenosis
AVR: Aortic valve replacement
CT: Computerised tomography
DICOM: Digital imaging and communication in medicine
EACTS: European Association for Cardio-Thoracic Surgery
ECG: Electrocardiogram
eCRF: Electronic case report form
ESC: European Society of Cardiology
IFU: Instructions for use
KCCQ: Kansas City Cardiomyopathy Questionnaire
MCST: Multi-slice computed tomography
NYHA: New York Heart Association
PPM: Patient prosthesis mismatch
QoL: Quality of life
SD: Standard deviation
SF-12: Short Form-12 Health Survey
SVD: Structural valve degeneration
